# Dermoscopic changes of tattoos over melanocytic nevi^☆^

**DOI:** 10.1016/j.abd.2023.08.015

**Published:** 2024-05-31

**Authors:** Felipe Miguel Farion Watanabe, Lia Dias Pinheiro Dantas, Renan Rangel Bonamigo

**Affiliations:** a Department of Dermatology, Hospital de Clínicas de Porto Alegre, Porto Alegre, RS, Brazil; b Postgraduate program in Medical Sciences, Universidade Federal do Rio Grande do Sul, Porto Alegre, RS, Brazil; c Department of Internal Medicine, Faculty of Medicine, Universidade Federal do Rio Grande do Sul, Porto Alegre, RS, Brazil

*Dear Editor*,

The prevalence of tattoos in the population has increased and dermatologists must be familiar with its complications. The survey carried out by Kluger et al. showed a prevalence of tattooed individuals in Brazil of 22.3%; the most prevalent age group being 25‒34 years old (30.3%).^1^ Bicca et al. analyzed the prevalence of tattoos in recruits in Pelotas, Rio Grande do Sul, and the result was 10.82% of 1,968 recruits.^2^

Nevi traumatized by tattooing may undergo changes that increase the suspicion of malignancy, such as cytological atypia, pagetoid spreading and dermal mitosis; therefore, nevi, pigmented lesions, or melanoma scars should not be tattooed. Tattooing pigment in nevi can either delay the diagnosis of malignant transformation or simulate findings of malignancy, resulting in unnecessary surgery. It is recommended to avoid tattooing areas of the body with an abundance of nevi, keeping a margin of 0.5 to 1 cm from these lesions. Patients with atypical nevi syndrome or a personal or family history of melanoma should consult a dermatologist prior to tattooing.^3^, ^4^, ^5^, ^6^.

A cross-sectional study was carried out, using convenience sampling between December 2021 and July 2022, at the Dermatology Outpatient Clinic of Hospital de Clínicas de Porto Alegre, in Porto Alegre, Rio Grande do Sul, Brazil. The inclusion criterion was having a tattoo. Exclusion criteria were being under 18 years of age or having some physical or psychiatric disability that made it impossible to understand the study and/or sign the Informed Consent Form. The tattoos were inspected and when they were over a nevus, the lesion was photographed and stored using a FotoFinder equipment. The dermoscopic characteristics were analyzed jointly by the researchers. This study was approved by the Ethics Committee under protocol number 52210121.2.0000.5327.

A total of 112 individuals participated in the research, who had 485 tattoos. Of these, 82 (16.9%) were located over melanocytic nevi, with a total of 194 nevi being tattooed. The study by Kluger retrospectively analyzed complications in 31 patients with tattoos and observed 10% tattooed nevi.^7^ This value is lower than that was found in the present study, which can be justified because this was a cross-sectional study in which all tattoos were inspected actively looking for nevi.

The following dermoscopic changes were observed in tattooed nevi: dots, globules, erythema, tattoo obliteration, amorphous blurring, blue veil, densification of the nevus pigment, partial obliteration of the nevus pigment, total obliteration of the nevus pigment, pseudonetwork and perifollicular pigment. Fig. 1, Fig. 2, Fig. 3, Fig. 4 demonstrate some aspects observed in the study.

**Fig. 1:**
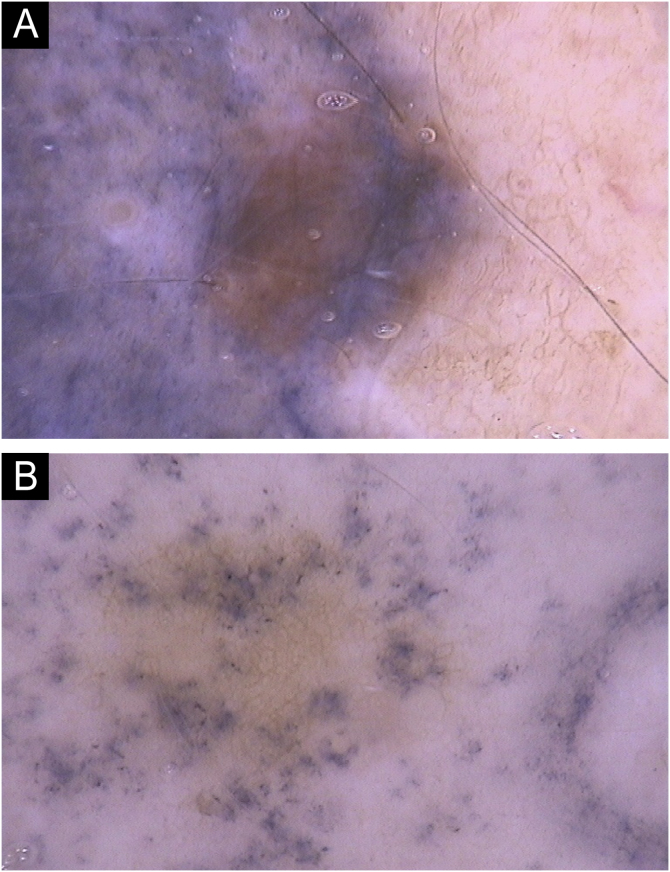
(A) Tattoo pigment in an amorphous blurring pattern with an area of densification of the melanocytic network on the left edge and an area of blue veil on the lower edge of the nevus. (B) Tattoo pigment in a pattern of dots and globules.

**Fig. 2:**
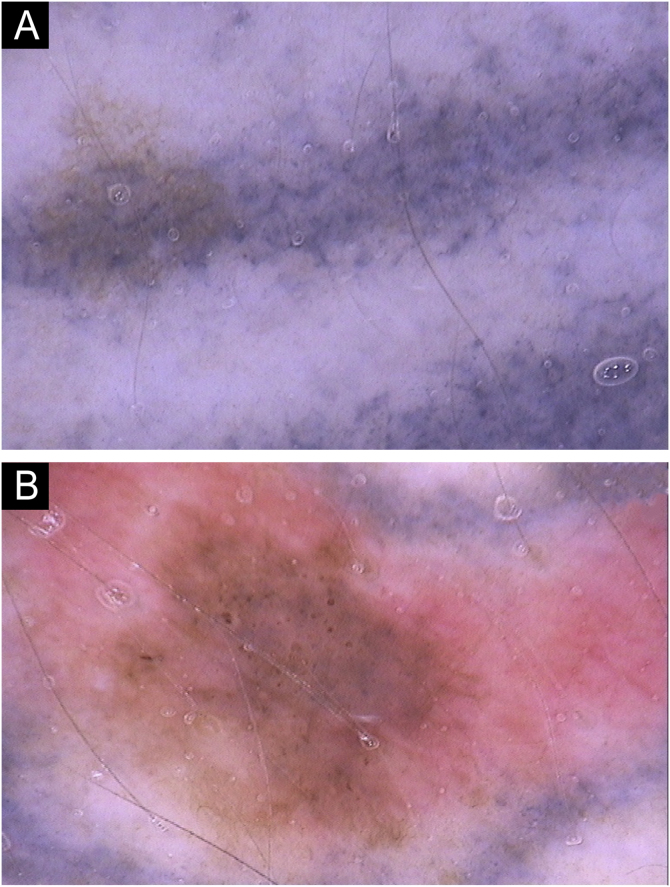
(A) Tattooing pigment with a lacy presentation characterized as a pseudonetwork. (B) Red tattooing pigment simulating erythema.

**Fig. 3:**
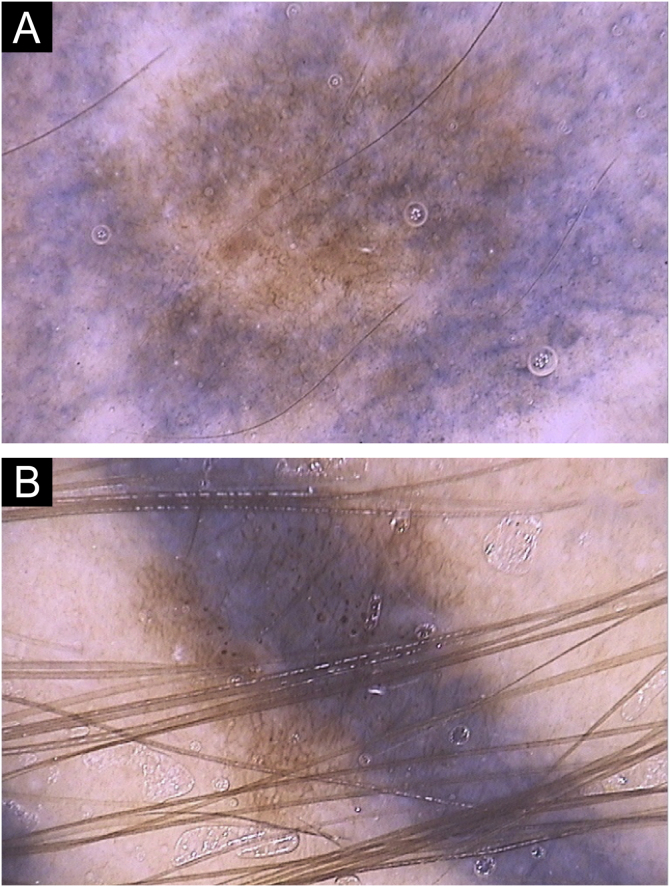
(A) Tattooing pigment causing partial obliteration of the nevus and (B) total obliteration of the nevus, in which the melanocytic network cannot be clearly observed in the tattooed area.

**Fig. 4:**
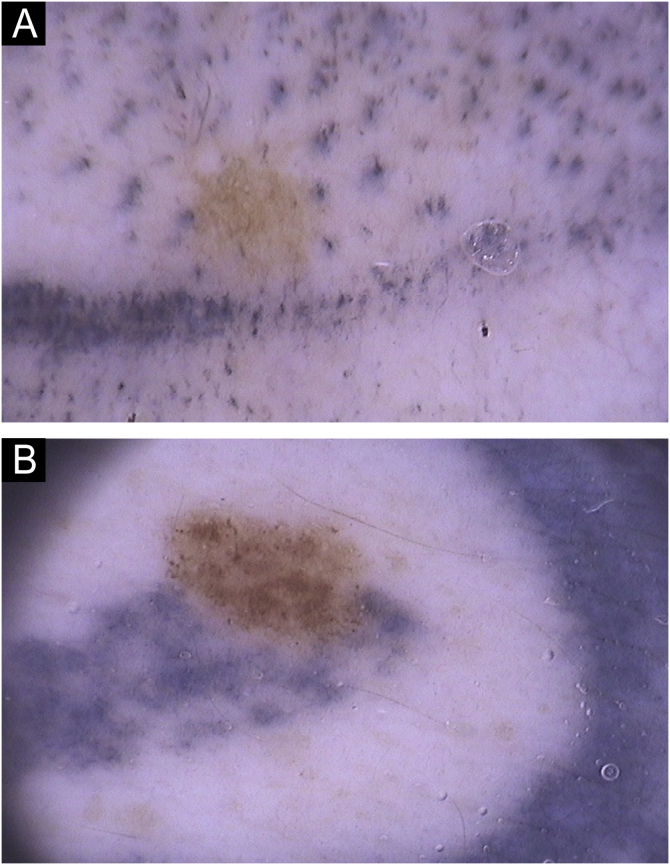
(A) Perifollicular concentration of tattooing pigment. (B) Nevus causing obliteration of the tattoo; melanin pigmentation extends over the tattoo. Abrupt interruption of the tattooing pigment is observed indicating the possibility of the nevus having appeared after tattooing.

Blurring was seen as amorphous dark areas in the nevus, and was the most observed change, present in 108 nevi (55.7%). Tattooing pigment present in small dots was present in 93 nevi (47.9%); while larger round clusters with the appearance of globules were present in 70 nevi (36.1%). Tattooing pigment present in small quantities in the nevus caused densification of the melanocytic network in 26 nevi (13.4%). An important observation is the blue veil ‒ a pattern attributed to malignant melanocytic lesions ‒ observed in 16.5% of the nevi in this study (Fig. 1).

The pseudonetwork pattern was seen as a lacy tattoo pigmentation in 30 nevi (15.5%). Erythema was observed in eight nevi (4.1%), and was present only in red pigment tattoos (Fig. 2).

Heavier tattooing caused obliteration of the nevus, attributed in this study as partial if it was still possible to observe the melanocytic pattern interspersed with the tattooing pigment, or total when it was only possible to observe the tattooing pigment (Fig. 3). These patterns were present in 44.3% and 21.1% of nevi respectively, and can hide asymmetries and other suspicious findings in nevi, which is a matter of concern, as together they were present in almost two-thirds of the nevi.

Perifollicular pigmentation was observed in only two nevi (1.0%). An interesting presentation, observed in 4.1% of nevi, was tattoo obliteration, in which the nevus stood out and there was an abrupt interruption of the tattooing pigmentation (Fig. 4). The authors believe this pattern may be associated with the appearance or growth of the nevus after the tattoo, and that histopathological evaluation could be informative.

On most occasions, the examiner can differentiate between changes caused by tattooing and changes due to the nevus. However, tattooing pigment can cause obliteration of the nevus, impairing the observation of the melanocytic network and consequently the screening for melanoma. To the best of the authors knowledge, this is the first study analyzing the dermoscopic patterns of nevi that have undergone tattooing.

## Financial support

None declared.

## Authors' contributions

Felipe Miguel Farion Watanabe: Design and planning of the study; collection, analysis and interpretation of data; drafting and editing of the manuscript; critical review of the literature.

Lia Dias Pinheiro Dantas: Design and planning of the study; effective participation in research orientation; approval of the final version of the manuscript.

Renan Rangel Bonamigo: Design and planning of the study; analysis and interpretation of data; effective participation in research orientation; approval of the final version of the manuscript.

## Conflicts of interest

None declared.
